# Autologous dental pulp mesenchymal stem cells for inferior third molar post-extraction socket healing: A split-mouth randomised clinical trial

**DOI:** 10.4317/medoral.22466

**Published:** 2018-06-21

**Authors:** Luis Barbier, Eva Ramos, Josu Mendiola, Olivia Rodriguez, Gorka Santamaria, Joseba Santamaria, Icíar Arteagoitia

**Affiliations:** 1 MD, PhD, Professor, Cruces University Hospital, BioCruces Health Research Institute, University of the Basque Country; 2 PharD, PhD, Pharmacist, BioCruces Heath Research Institute; 3 MD, Neuroradiologist, Cruces University Hospital, BioCruces Heath Research Institute; 4 MD, PhD, Associate Professor, University of the Basque Country, BioCruces Health Research Institute

## Abstract

**Background:**

Since the discovery of adult mesenchymal stem cells extensive research has been conducted to determine their mechanisms of differentiation and effectiveness in cell therapy and regenerative medicine.

**Material and Methods:**

To assess the efficacy of autologous dental pulp mesenchymal stem cells delivered in a collagen matrix for post-extraction socket healing, a single-centre, double-blind, randomised, split-mouth, controlled clinical trial was performed. Both impacted mandibular third molars were extracted from 32 patients. Dental pulp was collected and dissociated; the resulting cell suspension, obtained by centrifugation, was incorporated into a resorbable collagen matrix and implanted in 32 experimental post-extraction sockets. Collagen matrices alone were implanted in 32 contralateral, control post-extraction sockets. Two neuroradiologists independently assessed the extent of bone repair at 6 months after the extractions. Computed tomography (CT, Philips Brilliance) and an advanced display platform (IntelliSpace Portal) was used to record extraction socket density, expressed as Hounsfield units (HU) and height (mm) of the distal interdental bone septum of the second molar. Measurements at 6 months post-extraction were compared with measurements obtained immediately after extraction. Data were analysed with the statistical program STATA 14.

**Results:**

Two patients dropped out of the study. The final sample consisted of 22 women and 8 men (mean age, 23 years; range: 18–30 years). Clinical, radiological, and surgical characteristics of impacted third molars of the control and experimental groups were homogeneous. Measurements obtained by the two neuroradiologists showed agreement. No significant differences were found in the extent of bone repair during analyses of density (*p*=0.4203 neuroradiologist 1; *p*=0.2525 neuroradiologist 2) or interdental septum height (*p*=0.2280 neuroradiologist 1; *p*=0.4784 neuroradiologist 2).

**Conclusions:**

In our clinical trial, we were unable to demonstrate that autologous dental pulp mesenchymal stem cells reduce socket bone resorption after inferior third molar extraction.

** Key words:**Clinical trial, autologous, pulpal stem cells, extraction socket healing.

## Introduction

Since the discovery of adult mesenchymal stem cells (MSCs), extensive research has been conducted to determine their mechanisms of differentiation and effectiveness in cell therapy and regenerative medicine. MSCs can be isolated from several cell extracts, and some of the most accessible extracts are from the oral cavity (OMSCs). These have been isolated from deciduous teeth, dental follicle, apical papilla, periosteum, dental ligament (LMSCs) and dental pulp (PMSCs) (1-2). MSCs are multipotent stromal cells that can be safely cryopreserved, used with several scaffolds, and extensively proliferate. They have long lifespans and can build in vivo adult bone with Havers channels and the appropriate vascularisation (3). Importantly, they can avoid the isolation, differentiation, expansion, and proliferation required in previous cell culture methods, which were not compatible with clinical practice due to the extensive manipulation of pulp tissue and its lengthy preparation process.

To facilitate clinical management, the Rigenera Protocol (Human Brain Wave srl, Torino, Italy) allows the production of adult mesenchymal stem cells from a minimum quantity of connective tissue of adult dental origin. A medical device, Rigenera Machine®, and Rigeneracons® sterile filters can disaggregate dental tissue and perform the filtering necessary to promote the expulsion of older differentiated cells and the enrichment of younger progenitor cells present within LMSC (4) and PMSC (5) populations. In this approach, the dental tissue experiences a phase of collection, foll-owed by mechanical disaggregation, filtering of the MSC cell suspension, and immediate application to clinical practice (5). This technical procedure neither expands nor manipulates the resulting cells. They constitute autologous tissues, such that the patient can be both donor and recipient (6).

A large gap between basic and translational research publications is present in the literature regarding OMSCs, with very few reports of clinical trials and clinical cases of bone or periodontal repair with LMSCs (4), PMSCs (5,7,8), and MSC derivatives of periosteum (9). To characterise the regenerative potential of autologous dental pulp mesenchymal stem cells (ADPMSCs) with the Rigenera® Protocol, we designed an independent, randomised, split-mouth clinical trial. We compared the bone healing efficacy of ADPMSCs, delivered in a resorbable collagen matrix, when implanted in impacted inferior third molar (ITM) post-extraction sockets (experimental group, EG) against the bone healing response of the alveolar sockets of the contralateral impacted ITM in which only collagen matrix is implanted (control group, CG). We tested the null hypothesis that bone healing in the EG and CG behaves similarly, with a maximum difference in proportions of 0.4.

## Material and Methods

We designed an independent, single-centre, double-blind, randomised, split-mouth controlled clinical trial. The trial was approved by the Ethical Committee of Clinical Investigation of Cruces University Hospital (Protocol number EC20141) and registered on the EudraCT database (number: 2014-001913-18). It was conducted in compliance with current legislation, the ethical principles of the Declaration of Helsinki, and good clinical practice.

We included patients with ages ranging from 18 to 30 years who attended the outpatient clinic of the Department of Maxillofacial Surgery at our hospital. For all patients, bilateral extractions of impacted mandibular third molars for mechanical reasons were indicated.

Patients with systemic diseases, risk factors for bacterial endocarditis, recurrent oral diseases, septic mouth, an infectious process, or who had undergone antibiotic treatment within 10 days of extraction were excluded from the study. Allergic patients, patients with known intolerance to any study medication, and pregnant or lactating women were also excluded from the study. Patients who showed cysts in radiographic images of either ITM, and those with possible roots in the inferior dental canal, were also excluded. All excluded patients were removed prior to randomisation. Patients who chose to drop out of the clinical trial, as well as those who were not followed-up, were excluded post-randomisation.

Sample size was calculated prior to the start of the study with the statistical software STATA 14 (StataCorp LP, College Station, TX, USA). Based on previous research studies, we assumed that post-extraction physiological bone resorption may reach 50% (10); thus, we considered it clinically relevant to assess whether resorption could be reduced to 10% by using the proposed ADPMSC treatment. A sample of 30 patients was required to compare the hypothesis with a 1-type error of 0.05 and 80% power for a difference in proportions of ≥0.4, assuming possible losses of 10% (33 patients).

Consecutive patient sampling was performed. Patients who met the inclusion criteria were asked to sign the informed consent form in order to participate in the clinical trial. Extraction sockets assigned to the EG were treated with ADPMSC, prepared according to the Rigenera® Protocol, incorporated into an absorbable lyophilised collagen matrix Hemo-Klee® (Directive 93/42 EEC. Euroklee S.L. Cerdañola del Valles Barcelona, Spain), whereas those assigned to the CG were treated with collagen matrix alone. The decision of which socket (left or right) received the ADPMSC treatment was made with codes generated by an independent professional, who used randomisation software. The independent professional concealed the allocations in sealed and sequentially numbered envelopes. A maxillofacial surgeon (MS1) performed the standardised procedure for both third molar extractions. A second maxillofacial surgeon (MS2) prepared and implanted ADPMSCs in the EG and collagen matrix in the CG, then sutured the flaps. MS2, who did not participate in the analysis of the results, was the only non-blinded researcher in this study.

**Figure 1:**
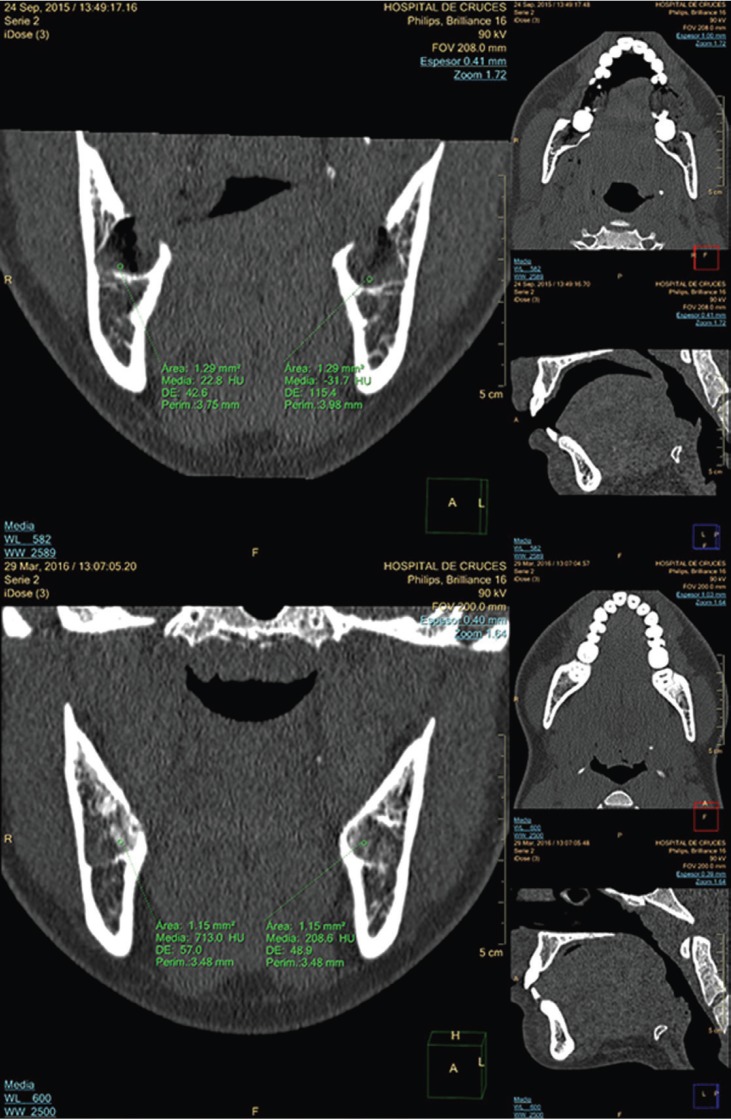
Top image t0 in multiplanar format, with windows for bone density assessment in intact post-extraction sockets. Average densities are measured in the lower half of both sockets. Bottom image t6, with alveolar repair on both sides.

To assess the bone repair achieved, at 6 months after ITM extraction, two CT scans were performed (Philips Brilliance 16; Philips HealthSystems, Amsterdam, Netherlands): one on the day of the surgery (t0) and the other 6 months later (t6). Socket density (in Hounsfield units, HU) and resorption response (in height, mm, of the interdental distal septum to the second molar) were assessed. Response variables were bone density (BDD) and bone resorption (BRS). BDD was assessed by subtracting the final average socket density (measured in a region of interest in the lower half of the socket at t6) from the initial average density measured in the same area at t0 (Fig. 1).

**Figure 2:**
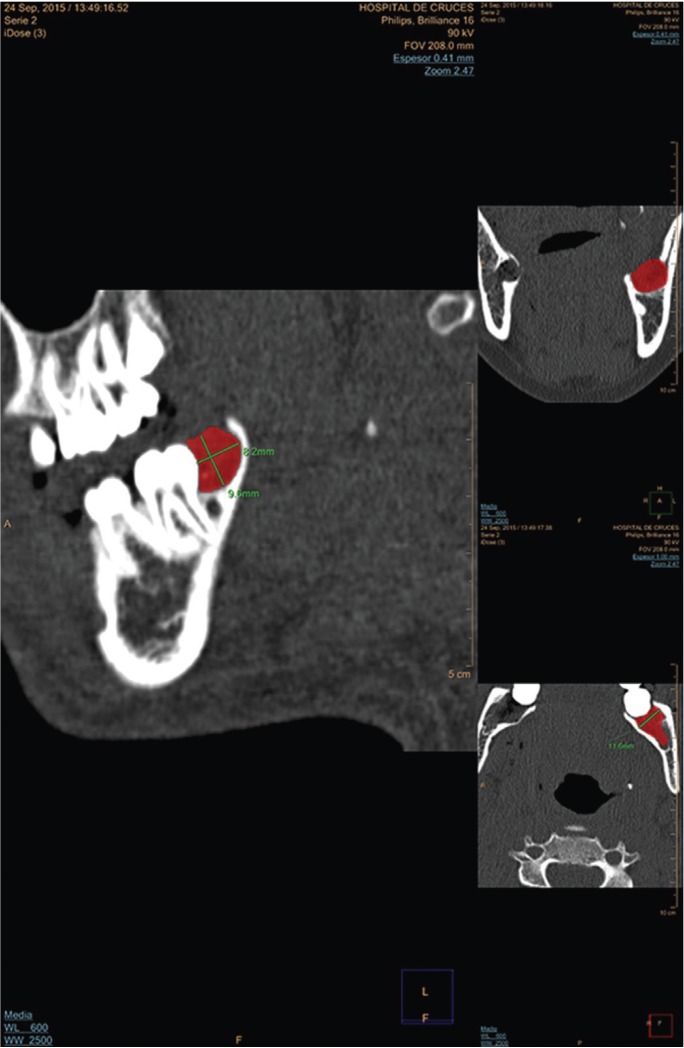
Results at day t0. Images in multiplanar format with windows for bone density assessment. Measurements of left post-extraction cavity were made in the three planes, represented by the area in red colour.

BRS was assessed by calculating the mean height (mm) of the interdental distal septum to the second molar at 6 months and the mean height recorded immediately after the extraction (Fig. 2).

-Procedure

During the first contact with patients who could be recruited for the clinical trial, MS1 studied possible candidates. Bilateral impaction of both ITM and gingival coverage (enabling flap closure without tensions) were confirmed radiologically and clinically, respectively. The inexistence of any possible reason for exclusion was assessed in the clinical history. Patients were provided with information regarding the surgical procedure and clinical trial. The informed consent form was explained to them and they were each provided with a copy.

During the second visit, the surgical procedures were performed after repeat confirmation that patients met the inclusion criteria and had signed the informed consent form. Patients were then assigned a sequential patient number. During this second appointment, we also recorded data regarding demographic, clinical, radiological, and surgical variables.

Demographic variables included age (corresponding to the age of the patient on the day of the intervention), sex (male or female), and ethnicity (Caucasian, Black, Asian, others) of patients. Clinical and radiological variables included the use of contraceptives (response: yes or no) in the last month, the use of psychotropic drugs (response: yes or no) and whether they were taken in the last month, and tobacco smoking (expressed as number of cigarettes per day at the time of the intervention).

After the local anaesthetic nerve block was performed, a millimetre periodontal probe (SM 13 Bontempi®) of thickness 0.5–11.5 mm was used to record the depth (mm) at three points distal to the second molar in both ITMs. The ITM was classified as type I when the crown showed no bone coverage on radiographic analysis and clinical analysis showed that the gingival coverage would allow flap closure without tensions; as type II when the crown was partly covered by bone and gum; and as type III when the third molars were fully covered by bone and gum.

The angulation and depth of the impacted mandibular third molars were determined by using the classifications of Pell & Gregory and Winter (11). All operations were performed by the same maxillofacial surgeon (MS1) with a standardised procedure for both third molars, beginning on the right side in all cases. The mouth was previously rinsed with for 20 sec with Lacer® 0.12% chlorhexidine mouthwash (Lacer S.A. Cerdañola del Vallés, Barcelona, Spain). Local and regional anaesthesia were performed with 3.6 ml articaine (Ultracain®; Normon S.A. Tres Cantos, Madrid, Spain) and epinephrine (40/0.01 mg/mL) for blocking inferior alveolar and buccal nerves. A full thickness mucoperiosteal flap was elevated and reflected. The incision was made with a No. 15 surgical scalpel blade, forming an envelope flap that extended from the mesial aspect of the second molar to the ramus, with lateral divergence of the posterior extension. Bone removal and tooth sectioning were performed by using a hand piece with a round bur and saline irrigation. After the extraction, the sockets were debrided and necrotic tissue was removed. The area was irrigated with 3 ml 0.12% Lacer® chlorhexidine and saline solution for 1 min (11).

MS1, who performed the extractions, did not consider which groups (EG or CG) patients belonged to. Subsequently, after extracting each ITM, MS2 placed the ITM in a tray with sterile gauze soaked in 0.12% chlorhexidine for 2 min. By using a round bur, hand piece, and saline irrigation, an indentation was made in the enamel-cementum line, which gradually became a groove. When the groove was sufficiently deep, odontosection of the molars was completed by pressing with a chisel, thereby providing access to the pulp chamber. The pulp from both pulp chambers was collected with a sterile curette and deposited in a Rigeneracons® capsule; then, 1 mL saline solution was added. The capsule was agitated for 1 min in the Rigenera® Machine. MS2 collected the PMSC suspension by aspiration with a syringe, incorporated it into the collagen matrix Hemo-Klee®, left it standing for 8 min, and implanted it in the left or right socket after opening the randomisation envelope. The collagen matrix was exclusively implanted contralaterally with 1 mL saline solution. Lastly, flap closure was performed with a 3/0 absorbable suture, ensuring complete flap closure to avoid alveolar permeability. An Augmentine® 875/125 mg tablet (GlaxoSmithKline S.A. Tres Cantos, Madrid, Spain) was prescribed to all patients 1 h before the extractions. They also received a prescription of one tablet every 8 h for another 5 days to limit possible infectious and inflammatory complications. All patients were administered Enantyum® 25 mg film-coated tablets (Menarini SA, Badalona, Barcelona, Spain) every 8 h until complete remission of pain, and performed rinsing with Lacer® 0.12% chlorhexidine mouthwash every 8 h for 7 days.

Surgical variables recorded include the surgical time, tooth sectioning, and ostectomy. Surgical time was measured by using a stopwatch from the first incision until the last suture was completed. Tooth sectioning was recorded as yes or no. No pulp cavity was invaded during the extraction. With regards to ostectomy, a score of 0 was assigned when ostectomy was unnecessary; 1/3 was assigned when ostectomy was necessary to uncover the crown; and 2/3 was assigned when ostectomy was performed to uncover the crown and part of the root.

Written postoperative instructions were distributed. A 24-hour telephone number and a clinical trial participation card were also provided. Immediately after the extractions, multi-slice CT scanning without contrast was performed with the Phillips Brilliance 16-slice CT scanner (Phillips, Amsterdam, Netherlands) at the Department of Radio-logy, with low energy, low power, and an exposure time of seconds with high image quality. The following technical characteristics were used: 1 mm slice thickness, 16 × 0.75 degree of collimation, 178 mm field of view (FOV), 512 × 512 matrix, 90 kV dose, 100 mAs, Level iDose 3, 6 mGy volume CT dose index (CTDI), according to the standard “As low as reasonably achievable.”ALARA. From this CT scan, two neuroradiologists, who did not know whether the socket was for a patient in the EG or CG, independently made the following measurements with the advanced analysis platform IntelliSpace Portal (Phillips, Amsterdam, Netherlands).

**Figure 3:**
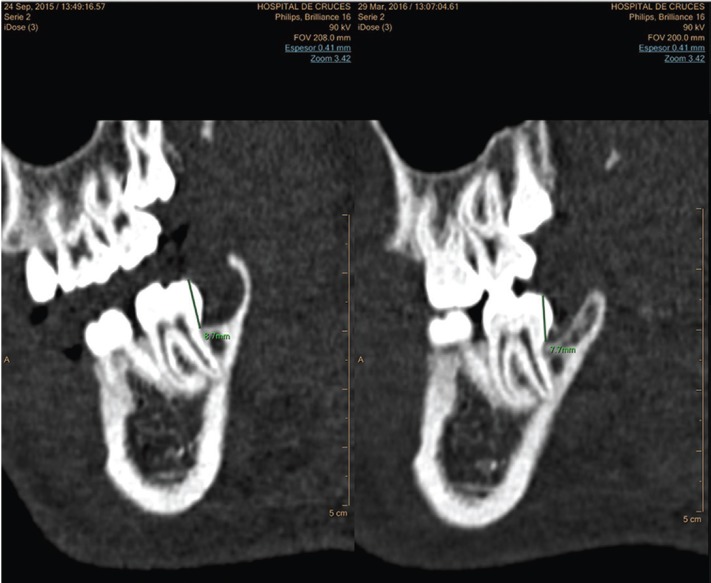
Distal interdental septum of the second molar at t0 and t6. Images acquired by using bone windows. Measurement of distal interdental septum height (mm) in second molars at the times t0 and t6.

1.- Residual volume of both extraction sockets (mm3) on the day of extraction t0: three diameters, namely the latero-lateral (LL), anteroposterior (AP), and craniocaudal (CC) diameters, were measured. The volume was calculated by using the formula LL × AP × CC/2 (Fig. 3). This volume was used to analyse the homogeneity between the CG and EG.

2.- Density (HU) of the residual sockets: this was assessed by using the mean of measurements taken in one region of interest (Fig. 1).

 3.- Bone resorption: This was assessed by measuring the height (mm) from the occlusal plane to the highest point of the interdental septum distal to the second molar (Fig. 2).

During the third appointment, at 6 months after the intervention, a second CT scan was performed, in the same manner as the original scan. Both neuroradiologists, who did not know which sockets were treated with the ADPMSC suspension, independently assessed the variable response, including the density (HU) of the regenerated alveolus and the bone resorption of the interdental septum distal to the second molar (mm).

The values of the clinical, demographic, radiological, and surgical variables of the 64 ITMs from the CG and EG were recorded in an Excel spreadsheet. The values assessed by each neuroradiologist for each measurement, immediately after the surgery (t0, volume, density, and height) and 6 months after the surgery (t6, density and height) were also recorded. Adverse events were recorded.

Quantitative variables were assessed by their mean values while qualitative variables were assessed by calculating absolute frequencies. The homogeneity of ITM characteristics from the CG and EG were assessed by chi-squared tests, Fisher’s exact tests, and analysis of variance, depending on the nature of the variables (qualitative or quantitative). Inter-observer agreements (between neuroradiologists 1 and 2) of variables (volu-me, density, and height) recorded immediately after the surgery, and of response variables BRS and BDD, were assessed by using the mean Kendall rank correlation coefficient. To analyse the response variables BRS and BDD, we assessed fit-to-normality with the Shapiro-Wilk Test. We tested the null hypothesis of no difference in reparative response between the EG and CG, by using the t-test when the variable followed a normal distribution, and by Mann-Whitney U test when the variable did not follow a normal distribution. Significance level was pre-set to 0.05. Stata14 (StataCorp LP, College Station, TX, USA) was the statistical software used.

## Results

Recruitment began in March 2015. The first patient underwent extractions in May 2015 and the last patient underwent extractions in February 2017. In total, 32 patients were recruited; two were excluded because they were unable to undergo a CT scan at 6 months after the extraction. The final sample consisted of 30 patients and 60 molars (30 in the EG and 30 in the CG).

Because this was a split-mouth study, demographic variables were identical for the ITMs from the EG and CG. The patients were Caucasian, eight men and 22 women. Their mean age was 23 years (range: 18–30 years). Seven patients were taking contraceptives, one was undergoing treatment with psychotropic drugs, four patients smoked < 10 cigarettes per day, and five smoked 10–20 cigarettes per day.

No significant differences in clinical, radiological, and surgical characteristics of ITMs were found between the CG and EG ( Table 1). We concluded that there was an agreement between the independent observations of the two neuroradiologists in the records analysed ( Table 2). We analyse the normality-adjusted values of response variables, BDD and BRS, assessed by both neuroradiologists. Bone repair, in terms of BDD and BRS, showed no significant differences between the control and experimental groups, either when analysing density (BDD: *p*=0.4203 neuroradiologist 1; *p*=0.2525 neuroradiologist 2) or when analysing the interdental septum height (BRS: *p*=0.2280 neuroradiologist 1; *p*=0.4784 neuroradiologist 2) ( Table 3).

**Table 1:**
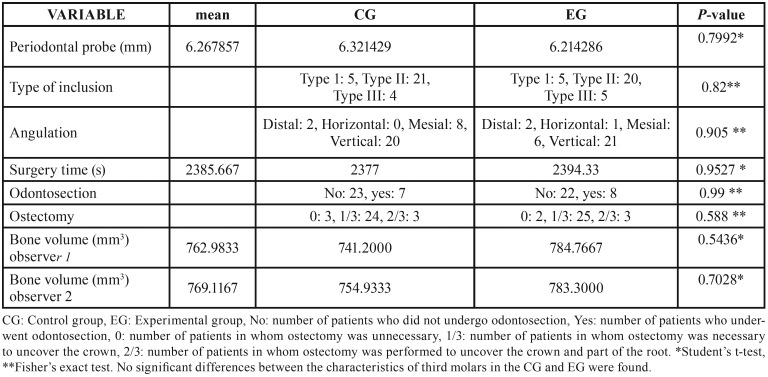
Homogeneity between study groups.

**Table 2:**
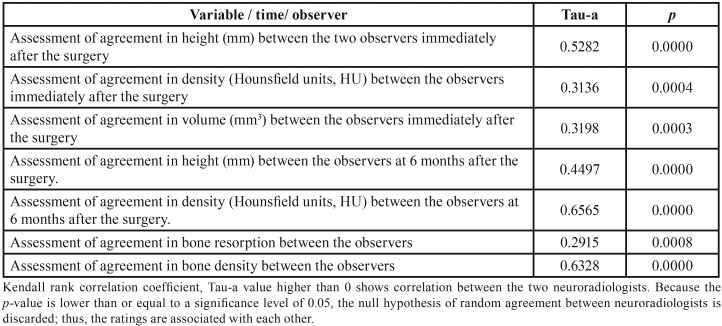
Inter-observer agreement in this study.

**Table 3:**
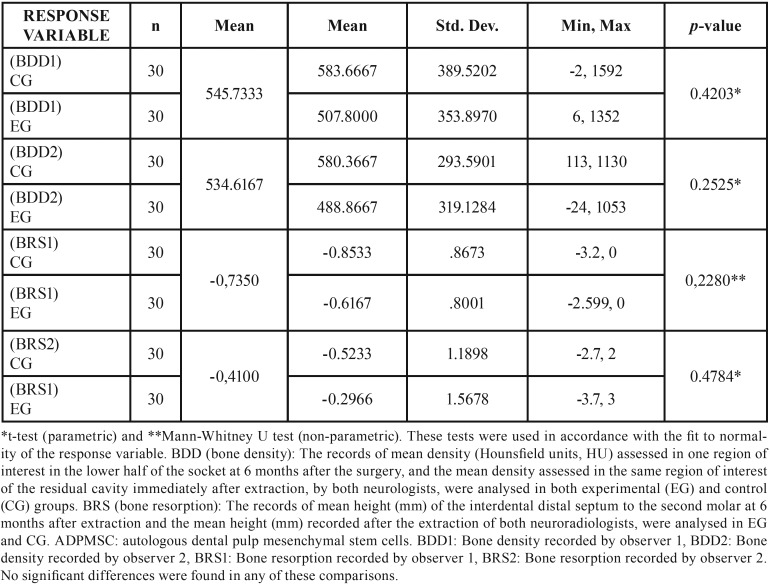
Analysis of the response variable, density, and height between the experimental group with autologous dental pulp mesenchymal stem cells (ADPMSCs) and the control group without ADPMSCs.

No morbidity was observed during the clinical trial, and no information was gathered from other studies that discouraged continuation of the trial.

## Discussion

This manuscript was written in accordance with the recommendations of the CONSORT 2010 Declaration (12). Our objective was to assess the effect of ADPMSCs on limiting socket bone resorption after ITM extraction.

Regenerative medicine aims to repair damaged tissues with cell therapy procedures. It is based on the use of stem cells from different origins; these cells can be differentiated into specific mesenchymal tissues by using different procedures. Adult dental pulp stem cells are one of the sources used in the repair and regeneration of bone and periodontal and dental defects. One of the limitations of application to clinical practice could be the prolonged time between the collection of dental pulp and isolation of PMSCs, which likely explains why few clinical trials are registered in the European Medicines Agency (www.ema.europa.eu) and in the National Institutes of Health of the United States of America (https://www.nih.gov).

In an experimental animal model, D’Aquino *et al.* de-monstrated the capacity of a suspension of digested dental pulp cells, cultured in a low-density liquid, to induce osteogenesis, angiogenesis, and vascularization of bone tissue (13). The same research team simplified the procedure by demonstrating the capacity to produce bone nodules, without the requirement for prior expansion of PMSCs, in an experimental animal model (14).

A controlled split-mouth clinical trial was published regarding bone repair in post-extraction sockets of the ITM in seven patients. To collect pulp tissue, the upper third molars were removed; pulp cells were mechanically dissociated, filtered, and cultured. After 21 days of culture, ADPMSCs were obtained and, by using a collagen matrix, these cells were implanted in the experimental post-extraction socket of the ITM; collagen alone was implanted in the control socket. Consequently, bone regeneration was higher by 25% in the experimental group (7).

A study conducted three years later on the same samples concluded that the regenerated bone in the ADPMSC group was evenly vascularised and more compact than that in the control group, where a collagen sponge alone was implanted (8). Following these preliminary studies, the Rigenera® Protocol was developed and applied clinically (4,5,6,9). ADPMSCs obtained from dental ligament-derived tissue were added to a collagen sponge and implanted in the post-extraction cavity of an ITM; the contralateral cavity was implanted with collagen alone. After 6 months, clinical beginning/ending differences were noted in probing at the experimental site (12 mm/3 mm), compared with the control site (11 mm/7 mm) (4). A report of a case where the sinus cavity was filled with grafted ADPMSCs indicated that the density of the newly formed bone was approximately twice that of the native bone (5). Application of the protocol to adult periosteal tissue demonstrated that progenitor cells for use in tissue regeneration could be produced (6). In a subsequent controlled multi-centre clinical trial of 35 patients, a suspension of autologous periosteal progenitor cells was grafted with a collagen matrix in the post-extraction socket; outcomes in these patients were compared with those in a control group where collagen alone was used. That study demonstrated that the autologous periosteal progenitor cells accelerated ossification and reduced bone reabsorption in the alveolar crest, thus preserving the socket (9).

By using the Rigenera® Protocol, we have analysed the ability of ADPMSCs to limit bone resorption in a model of ITM extraction, which is frequently used to assess the efficacy of anti-inflammatory and analgesic medicines. For this purpose, we conducted an independent clinical trial with a double-blind design, strict randomisation, and analysis of the inter-observer agreement of response variables, thereby providing solid internal validity. The image-measuring instrument used was the advanced display platform IntelliSpace Portal, which generates actual measurements of bone density and interdental septum height.

Recruitment was slower than expected due to the need to extract ITMs from each of the patients who had similar clinical and radiological characteristics. We recruited young patients aged between 18 and 30 years, from a healthy population, to minimise a possible effect of a decreased frequency of mesenchymal stem cells with age (15). When third molars are included, the pulp collected from the pulp chamber is, theoretically, free of disease. The split-mouth model allowed each patient to have control. The ADPMSC suspension was delivered in a collagen matrix, which is the most commonly used vehicle in this type of research (16).

The limitations of our clinical trial include the narrow age range, from 18 to 30 years. Furthermore, we did not perform immunohistochemical studies on the newly formed tissue, and we only evaluated the results once, at 6 months after surgery. A recent systematic review found some standardisation gaps in the method of isolation of stem cells from the oral cavity and stated that methodological heterogeneity should be assessed to create a protocol standardising their isolation and differentiation (2). We agree with the findings of the systematic review.

Within the context of the aforementioned limitations, we were not able to demonstrate that autologous dental pulp mesenchymal stem cells have the ability to limit socket resorption after impacted lower third molar extraction.

Source of Funding: Project PI13/00541, funded by Instituto de Salud Carlos III and co-funded by European Union (ERDF/ESF, “Away to make Europe/Investing in your future”).
